# Oxidative Stress and Inflammation in Renal and Cardiovascular Complications of Diabetes

**DOI:** 10.3390/biology10010018

**Published:** 2020-12-30

**Authors:** Amelia Charlton, Jessica Garzarella, Karin A. M. Jandeleit-Dahm, Jay C. Jha

**Affiliations:** 1 Department of Diabetes, Central Clinical School, Monash University, Melbourne 3004, Australia; aecha7@student.monash.edu (A.C.); jsgar2@student.monash.edu (J.G.); karin.jandeleit-dahm@monash.edu (K.A.M.J.-D.); 2 Institute for Clinical Diabetology, German Diabetes Centre, Leibniz Centre for Diabetes Research at Heinrich Heine University, Dusseldorf 40225, Germany

**Keywords:** diabetes mellitus, diabetic complications, reactive oxygen species, inflammation, cardiovascular disease, diabetic kidney disease

## Abstract

**Simple Summary:**

The progressive nature of type 2 diabetes mellitus (T2DM) leads to micro- and macro-vascular complications, including renal and cardiovascular disease. These alone, or in combination, are a major cause of premature morbidity and mortality in diabetic patients. Despite advances in glucose lowering treatments, these diabetic complications are still inadequately prevented or reversed. This ongoing cardiovascular–renal burden in diabetes poses a heavy cost on the health care system. Therefore, there is an urgent need to develop more effective treatments. In this review, we discuss how oxidative stress and inflammation induce and perpetuate the renal and cardiovascular complications of diabetes. It is particularly important to understand these driving mechanisms in order to elucidate pharmacological targets and mechanism-based future drug therapies.

**Abstract:**

Oxidative stress and inflammation are considered major drivers in the pathogenesis of diabetic complications, including renal and cardiovascular disease. A symbiotic relationship also appears to exist between oxidative stress and inflammation. Several emerging therapies target these crucial pathways, to alleviate the burden of the aforementioned diseases. Oxidative stress refers to an imbalance between reactive oxygen species (ROS) and antioxidant defenses, a pathological state which not only leads to direct cellular damage but also an inflammatory cascade that further perpetuates tissue injury. Emerging therapeutic strategies tackle these pathways in a variety of ways, from increasing antioxidant defenses (antioxidants and Nrf2 activators) to reducing ROS production (NADPH oxidase inhibitors and XO inhibitors) or inhibiting the associated inflammatory pathways (NLRP3 inflammasome inhibitors, lipoxins, GLP-1 receptor agonists, and AT-1 receptor antagonists). This review summarizes the mechanisms by which oxidative stress and inflammation contribute to and perpetuate diabetes associated renal and cardiovascular disease along with the therapeutic strategies which target these pathways to provide reno and cardiovascular protection in the setting of diabetes.

## 1. Introduction

Type 2 diabetes mellitus has reached epidemic proportions, representing one of the most significant public health concerns of the 21st century [1]. The latest data published by the International Diabetes Federation in 2019 report that 463 million adults were living with diabetes—a number projected to reach a staggering 700 million by 2045 [2]. In Australia alone, approximately 1.7 million individuals suffer from diabetes and its subsequent complications, with T2DM representing 85–90% of all diabetic cases [3]. In 2019, diabetes and its complications were estimated to be responsible for the death of 4.2 million adults worldwide, equivalent to one death every eight seconds [2]. Moreover, the increasing prevalence of T2DM in young adults is of great concern as it will further affect the future global burden of diabetes, with a disproportionate impact on high-risk groups, such as Australian Indigenous populations [4].

The chronic hyperglycemic state of T2DM leads to the development of widespread and severe complications, which alone or in combination are a major cause of premature morbidity and mortality in diabetic patients [5]. As T2DM progresses, the toxic effects of the hyperglycemic environment induce pervasive organ damage, particularly impacting the vascular system [6]. Several vascular complications ensue, including those at a microvascular level, damaging kidneys (nephropathy) and eyes (retinopathy), or at a macrovascular level including atherosclerosis and cardiovascular disease [6,7]. All of these complications impair regular body function and decrease quality of life leading to premature death. The current management guidelines for T2DM focus on blood glucose stabilization combining education, lifestyle adjustments and pharmacotherapy, whilst monitoring for the aforementioned complications [8]. Despite these approaches, disease burden remains high with many patients still developing severe complications, not only placing a substantial load on individuals with the disease, but also the healthcare system as a whole [9]. As a result, the focus of current scientific research involves understanding the complex molecular mechanisms underlying the complications of T2DM. These endeavors aim to facilitate the development of new therapeutic strategies for the prevention and treatment of diabetic complications. Over the last decade, one such area of growing interest is the role of oxidative stress and inflammation. It has become well established that chronic hyperglycemia induces oxidative stress and inflammation, together driving the development and progression of T2DM complications, including renal and cardiovascular disease [10].

The state of oxidative stress refers to an imbalance in the production of reactive oxygen species (ROS) and antioxidant activity in the body, leading to an accumulation of ROS which directly damage cells and tissues [11]. ROS themselves are energized and reactive small molecules derived from oxygen, examples of which include superoxide (·O2), peroxyl (ROO·), hydroxyl (·OH), and hydrogen peroxide (H_2_O_2_) [12]. In addition, oxidative stress induces the activation of pro-inflammatory cytokines and subsequent inflammation which further promotes the production of ROS, thereby damaging cells and tissues [13]. At a cellular level, the mechanisms by which ROS production is enhanced include the polyol, hexosamine and protein kinase C (PKC) pathways, the NADPH oxidase family and/or via accumulation of advanced glycation end products (AGEs) [14]. Chronic hyperglycemia stimulates the activity of the aforementioned pathways, thus leading to elevated ROS production, contributing to renal and cardiovascular injury [15]. This review summarizes current research concerning oxidative stress and inflammation—particularly, how they interact and lead to renal and cardiovascular complications of diabetes. This is particularly relevant to pave the way for the development of novel pharmacological agents in order to alleviate the increasing burden of T2DM. 

## 2. Diabetes and Renal Complications: Oxidative Stress and Inflammation

Diabetic kidney disease (DKD) is the major microvascular complication of diabetes and the leading cause of renal failure worldwide, with up to 40% of diabetic patients developing renal disease, thus warranting further attention [16,17]. DKD is characterized by a progressive rise in albuminuria and the formation of glomerular lesions, leading to a gradual decline in the glomerular filtration rate (GFR) [18]. Additionally, the abnormal reduction in resistance of the afferent arteriole and elevation in that of the efferent arteriole lead to intraglomerular hypertension and additional kidney damage, which ultimately leads to end-stage renal disease (ESRD) [12]. The morphological changes occurring in DKD include thickening of the glomerular and tubular basement membranes, mesangial expansion, formation of microaneurysms, extracellular matrix accumulation, and glomerular and tubular cell injuries, leading to glomerulosclerosis and tubulointerstitial fibrosis, culminating in the progressive increase in albuminuria and gradual decline of kidney function [16,19,20]. Chronic hyperglycemia, dyslipidemia, and hypertension, as well as the activation of proinflammatory mediators and elevated ROS drive the development and progression of DKD, resulting in ESRD, leaving patients in need of dialysis and/or transplantation [16].

In a physiological context, several mechanisms are associated with the generation of renal ROS, including xanthine oxidase, NO synthase, the mitochondrial respiratory chain, and the NADPH oxidase (Nox) enzymes [12]. Growing evidence suggest that the members of the pro-oxidant Nox family, particularly Nox4 and Nox5 isoforms, are the key contributors of renal ROS generation in diabetes [16]. Indeed, increased expression of renal Nox4 and Nox5 have been demonstrated in the human diabetic kidney, as well as in renal cells, in response to high glucose and diabetes-induced AGE accumulation, angiotensin II and TGF-β [12]. Recent studies suggest that Nox5, either alone or together with Nox4, is predominantly responsible for the pathogenic renal ROS production in diabetes [16]. One such study revealed the pathogenic role of Nox4 in a murine model of DKD, whereby deletion of Nox4 provided partial renoprotection, evidenced by reduced albuminuria and attenuated structural abnormalities, including reduced mesangial expansion, glomerulosclerosis, and extracellular matrix deposition via reduction in ROS and inflammation of the kidney [21,22]. Similarly, pharmacologic inhibition of Nox4, with the first in class Nox1 and Nox4 inhibitors, GKT137831 (Setanaxib), displayed renoprotection in diabetic mice [21]. In the context of human pathology, it appears that Nox5 is upregulated in the diabetic kidney and is the main source of renal ROS, indicating the key role of Nox5 in human DKD [23,24]. Nox5 is expressed in humans but not in rodents, and hence has not been studied in conventional murine models of DKD. However, experimental studies using humanized Nox5 transgenic mice models demonstrate accelerated renal injury in diabetes, mimicking the human situation where Nox5 is a critical mediator in the pathogenesis of DKD [24,25]. In light of this, a study by Holterman et al. demonstrated that the podocyte-specific expression of human Nox5 in a mouse model of diabetes resulted in increased albuminuria and renal injury [23]. In addition, studies by our group have demonstrated that expression of Nox5 either in vascular smooth muscle cells or in endothelial cells leads to increased ROS production and an ensuing increase in mesangial expansion, glomerulosclerosis, fibrosis, and inflammation of the kidney [24]. Furthermore, in vitro studies have shown that high glucose increases Nox5 expression in renal cells and that silencing of Nox5 attenuates the hyperglycemia-induced increased expression of markers of inflammation and fibrosis via a reduction in ROS formation [24].

In DKD, inflammation occurs both systemically and localized to the kidneys with patients exhibiting increased plasma concentrations of pro-inflammatory cytokines, such as interleukins IL-1 and IL-6, tumor necrosis factor-α (TNF-α), and monocyte chemotactic protein-1 (MCP-1) [16]. In a diabetic milieu, exposure of renal cells to macrophages leads to the development of a proinflammatory state, which contributes to structural damage within the kidneys [26]. In addition, alterations in synthesis of prostanoids in diabetes also contribute to renal pathology through changes in renal hemodynamics. As a part of the inflammatory response, IL-1 and TNF- are expelled from renal macrophages, leading to increased permeability of endothelial cells and subsequent alterations in the hemodynamics of the kidney and a reduction in renal prostanoids, PGE_2_ synthesis. The decline in PGE_2_ levels in diabetes is crucial in the progression of DKD, where inflammation propagates renal injury [26].

**Figure 1:**
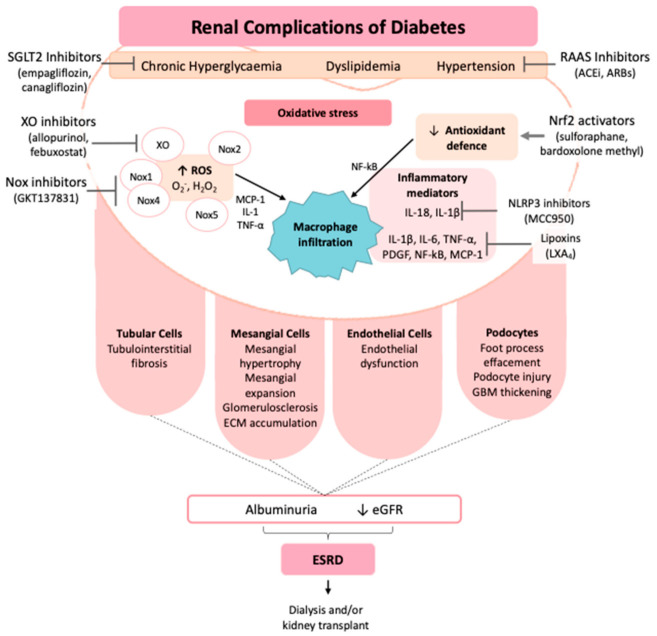
Pathophysiology and therapeutic strategies of diabetic kidney disease. ACEi, angiotensin-converting-enzyme inhibitors; ARBs, angiotensin receptor blockers; eGFR, estimated glomerular filtration rate; ESRD, end-stage renal disease; H_2_O_2_, hydrogen peroxide; IL, interleukin; MCP-1, monocyte chemotactic protein-1; NF-κB, nuclear factor-κB; NLRP3, NLR family pyrin domain containing 3; Nox, NADPH oxidase; Nrf2, nuclear factor erythroid 2–related factor 2; O^-^_2_, superoxide; PDGF, platelet-derived growth factor; RAAS, renin–angiotensin–aldosterone system; ROS, reactive oxygen species; SGLT2, sodium–glucose co-transporter 2; TNF-α, tumor necrosis factor alpha; XO, xanthine oxidase.

In normal physiological conditions, ROS are associated with the signaling in several cell types, including renal cells, and are involved in cell proliferation, differentiation, and apoptosis [12]. However, in pathological conditions, including T2DM, the increased formation of ROS stimulates inflammatory cells, leading to the production of inflammatory molecules, which exert a major role in the progression of renal damage [16]. Increased macrophage infiltration, often initiated by MCP-1, has a key role in the development of renal injury [12]. MCP-1 is expressed in renal endothelial, mesangial and monocytic cells and is closely regulated by TNF-α and IL-1, as well as being stimulated by ROS in diabetes [12]. Moreover, NF-κB is activated by oxidant/antioxidant imbalances and is considered the major driver of the inflammatory response occurring in DKD [12]. In a diabetic mouse model, increased renal expression of NF-κB was shown to be associated with activation of adhesion molecules and pro-inflammatory markers, including the aforementioned MCP-1, TNF-α, and IL-6, all of which are involved in the progression of DKD [12]. The expression of protein kinase C, particularly the PKC-*α* isoform, is increased in the diabetic kidney and found to be associated with renal inflammation and fibrosis [27]. In addition, our group and others have demonstrated that both Nox4 and Nox5 are involved in the regulation of PKC-*α* in DKD, suggesting the importance of these two critical pro-oxidant enzymes in the pathogenesis of DKD [28]. Furthermore, it has been identified that early growth response-1 (Egr-1) is a crucial intermediary transcriptional factor involved in inducing inflammation and fibrosis in DKD and is regulated by the ROS producing enzyme Nox4 [29]. Moreover, our unpublished data also suggest the regulation of Egr-1 by Nox5 in DKD. This evidence suggests a vicious bi-directional link between oxidative stress and inflammation in mediating renal injury in diabetes, as illustrated in Figure 1. 

## 3. Therapeutic Approaches for Diabetic Kidney Disease

### 3.1. Renin–Angiotensin–Aldosterone (RAAS) Inhibitors

T2DM is often associated with systemic hypertension, which can lead to increases in glomerular size and filtration rate, resulting in a decline in blood flow to the glomerulus and subsequent ischemic renal injury [30]. A plethora of evidence suggests a strong association between blood pressure control and a reduction in the development and progression of diabetic complications including DKD [16]. The renin–angiotensin–aldosterone system (RAAS) plays a key role in the pathogenesis of diabetic microvascular damage by evoking inflammation, oxidative stress and hemodynamic factors such as hyperfiltration. Current therapeutic strategies such as angiotensin converting enzyme (ACE) inhibitors and angiotensin-I receptor blockers (ARBs) target the RAAS and provide a degree of renoprotection in diabetes [31]. In DKD, the inhibition of RAAS has been associated with reduced AGE accumulation, TGF-β and Nox activity via a reduction in ROS and inflammation, thereby preventing the development of albuminuria, mesangial expansion and glomerulosclerosis [16]. In several studies, T2DM patients with DKD administered with lisinopril (an ACE inhibitor) and irbesartan (an ARB) displayed reductions in urinary MCP-1 excretion and DKD progression, as well as improved renal function [16].

### 3.2. SGLT2 Inhibitors

More recently, a newly introduced class of anti-diabetic drugs known as sodium–glucose co-transporter 2 (SGLT-2) inhibitors have displayed renoprotective properties through reducing elevated glomerular filtration, inflammation and oxidative stress [32]. In patients with T2DM, SGLT-2 inhibitors work by decreasing the renal threshold for glucose (RT_G_), thus increasing the urinary excretion of glucose from the proximal tubule, providing a degree of glycemic control and decrease in systemic blood pressure [33]. Moreover, it has been suggested that SGLT-2 inhibitors provide additional direct renoprotection complementary to inhibition of RAAS with ACE inhibitors and ARBs [34]. In two trials separately investigating the SGLT-2 inhibitors empagliflozin and canagliflozin, the progressive decline in kidney function observed in advanced DKD was slowed [34,35,36]. Empagliflozin attenuates the renal reabsorption of glucose in the proximal tubule, increasing the output of urinary glucose, thus reducing diabetes-induced hyperglycemia [35]. In T2DM, administration of empagliflozin leads to decreases in glycated hemoglobin, weight and blood pressure [35]. Similarly, canagliflozin exhibits renoprotective properties, hypothesized to be as a result of interactions with the hemodynamics of the kidney, leading to a decrease in glomerular hyperfiltration [33]. Additionally, phlorizin, a non-specific SGLT-2 inhibitor, has exhibited beneficial effects in STZ-induced diabetic rat models, reducing renal hyperfiltration and oxidative stress [37]. Although SGLT-2 inhibitors provide an effective treatment for T2DM not all patients are compatible with SGLT-2 inhibition. In patients who have progressed to severe kidney dysfunction and display a reduced GFR < 45 mL/min/1.73 m^2^, SGLT-2 inhibitors are contraindicated [38]. In addition, patients who receive SGLT-2 inhibitors are subject to a four-fold increase in their susceptibility to genital and urinary infections [39].

Whilst these current T2DM treatments are effective in slowing the progression of DKD, they fail to arrest or prevent renal injury [40]. Given the accumulating evidence identifying the involvement of oxidative stress and inflammation in the development of such complications and the lack of effective methods of disease prevention, emphasis should be placed on identifying the mechanism by which the effect of these processes can be reduced and reversed [41].

### 3.3. Therapeutic Approaches Targeting Oxidative Stress in Diabetic Kidney Disease

#### 3.3.1. Antioxidants

For the most part antioxidant treatments, including vitamin E and C, are yet to yield beneficial results in diabetic patients [42]. This is somewhat due to the lack of understanding towards the specific mechanisms underlying the function of antioxidants and the modality by which they should be administered to patients [16]. It has been suggested that identification of a therapeutic approach which can target the precise source of renal ROS in DKD may pose benefits over the use of non-specific systemic antioxidants in establishing renoprotection from oxidative stress [16].

#### 3.3.2. Nrf2 Activators

The cells of the human body possess their own innate defense strategy by which they fight against the development of oxidative stress. NFE2-related factor 2 (Nrf2) is a transcription factor and master regulator of an array of detoxifying genes which are activated in response to elevations in ROS [43]. When activated, Nrf2 induces the transcription of these genes which in addition to detoxifying ROS accumulation, remove damaged cell components and promote cell survival [43]. As such, Nrf2 upregulates the expression of various antioxidants such as catalase, glutathione, heme oxygenase-1, superoxide dismutase, and NADPH quinone reductase [16]. In Nrf2 deficient mice, the reduced expression of antioxidative genes leads to a heightened state of oxidative stress and subsequent activation of pro-inflammatory signaling [16,44]. Additionally, the overexpression of Nrf2 in renal endothelial cells resulted in the reduced expression of pro-inflammatory molecules including TNF-α, IL-1β, MCP-1, and VCAM1 [16,44]. Previous studies have shown the effect of Nrf2 activators including sulforaphane and cinnamic aldehyde in diabetic mice, whereby an increase in detoxifying enzymes leads to preservation of renal function and reduced renal damage, elucidating a therapeutic role for Nrf2 activators in DKD [45]. In a recent clinical trial termed BEACON, bardoxolone methyl was investigated as an activator of Nrf2. In phase 2 trials, administration of bardoxolone methyl in patients with chronic kidney disease (CKD) led to improved renal function. Phase 3 trials investigated the efficacy of bardoxolone methyl in the activation of Nrf2 in patients with T2DM, and displayed its ability to delay the progression of kidney disease to ESRD [46]. However, in patients with T2DM and stage 4 diabetic kidney disease the study also revealed a significantly higher rate of cardiovascular events in patients administered bardoxolone methyl in comparison with those who received the placebo, and thus the clinical trial was terminated. 

#### 3.3.3. NADPH Oxidase (Nox) Inhibitors

Previous research has identified several non-specific NADPH oxidase inhibitors including diphenylene iodonium, plumbagin, and apocynin which whilst partially inhibiting Nox also exhibit significant off-target effects [47]. Currently, the only Nox-specific inhibitors which have yielded beneficial results at the preclinical stage are GKT136901 and GKT137831, which both exhibit inhibition of Nox isoforms Nox1 and Nox4 [16]. In mouse model of diabetes, administration of GKT136901 decreased the production of hyperglycemia-induced ROS within the proximal tubules and reduced the extent of albuminuria [48,49]. Moreover, the administration of GKT137831 in diabetic mice revealed renoprotective and anti-atherosclerotic properties as evidenced by attenuation of renal ROS generation, inflammation and albuminuria [16,21]. Additionally, the Nox1 and Nox4 inhibitor GKT137831 is less active toward Nox2, which is a crucial contributor to immune defense [50]. Within diabetic murine models it is well established that GKT137831 exerts renoprotective properties via the inhibition of Nox4, warranting clinical investigation into its effect in diabetic patients. However, a 2013 short-term phase 2 study assessing GKT137831 in a population with T2DM and albuminuria demonstrated a reduction in inflammation but failed to show improvements in renal function [51]. Informed by several methodological shortcomings of this study, such as recruitment of patients with late stage DKD, short treatment period and low drug dosage, a 2020 phase 2 trial is being undertaken in patients with type 1 diabetes mellitus and kidney disease [52]. Despite this, further investigation is required into the effects of GKT137831 on Nox5 inhibition, which is important in the human context. Given the recent discovery of Nox5 in the human genome and its role in DKD, further investigation into Nox5 is required to offer impetus for the development of a Nox5-specific inhibitor. 

### 3.4. Therapeutic Approaches Targeting Inflammation in Diabetic Kidney Disease

#### 3.4.1. XO Inhibitors

In addition to anti-diabetic drugs, a growing interest in the role of chronic inflammation in the pathogenesis of DKD has led to the investigation of anti-inflammatory agents. Several anti-inflammatory agents may possess beneficial results in patients of DKD. Xanthine oxidase (XO) is an enzyme involved the development of hyperuricemia, which refers to abnormally high concentrations of uric acid, leading to excessive production of ROS and inflammation [53]. XO inhibitors are a group of anti-inflammatory compounds including allopurinol which is used to treat hyperuricemia induced gout [53]. To reduce levels of uric acid, the use of allopurinol in clinical trials revealed reductions in inflammation which led to reduced proteinuria and a deceleration in the decline of kidney function in diabetic patients [54]. Moreover, Febuxostat, a new non-purine selective XO inhibitor has also demonstrated beneficial results in patients with chronic kidney disease exhibiting the reduction of uric acid levels and slowing the decline in eGFR [55]. Additionally, another study investigating Febuxostat in patients with DKD and hyperuricemia revealed decreases in uric acid levels and stabilization of the GFR [56]. However, the difference in eGFR between the group administered Febuxostat and those who received the placebo was not significantly different and Febuxostat failed to attenuate the level of proteinuria. These findings suggest the use of Febuxostat in DKD requires further investigation.

#### 3.4.2. Lipoxins

In T2DM, the failure to resolve inflammation is considered pivotal in the progression of disease and the development of complications including DKD [57]. Lipoxins are a class of eicosanoids and endogenous anti-inflammatory molecules which in a physiological setting are synthesized at a low concentration [57,58]. In a pathological milieu, inflammatory stimuli significantly enhance the secretion of lipoxins, initiating their modulation of inflammation [58]. Lipoxin A4 (LXA_4_) is one such molecule synthesized during acute inflammation which exerts anti-inflammatory properties via interactions with formyl peptide receptor 2 (AXL/FPR2), a G-protein coupled receptor [57]. Studies investigating the potential therapeutic role for LXA_4_ in DKD reveal that intraperitoneally delivered lipoxin injections reduce the onset of diabetes-associated albuminuria, mesangial expansion and extracellular matrix accumulation in a murine model [57]. It is thought that lipoxins achieve this via the suppression of pro-inflammatory biomarkers including TNF-a, TGF-b, IL-1b, PDGF, and NF-kB [57]. More recently, an in vitro study has suggested the ability of LXA_4_ to attenuate the development of oxidative stress via the inhibition of the uric acid-associated activation of NADPH oxidase and subsequent ROS production [59]. The proposed underlying mechanisms by which this occurs implicates the inhibition of p47phox translocation from the cytoplasm to the membrane and subsequent attenuation of NADPH oxidase synthesis [59]. Further investigation utilizing in vivo subjects is required to determine the relevance and applicability of such findings for therapeutic purposes. 

#### 3.4.3. Other Agents Targeting Inflammation

Another anti-inflammatory compound used in the treatment of several diseases is a selective molecule inhibitor of the NLRP3 inflammasome referred to as MCC950, responsible for the downregulation of IL-1β production [60]. In a study in hypertensive mice, MCC950 reduced systemic blood pressure, as well as vascular dysfunction, inflammation and fibrosis of the kidney [61]. Another study in diabetic mice revealed that the MCC950 inhibition of the NLRP3 inflammasome reduced caspase-1 and IL-1β production and attenuated renal injury, suggesting that MCC950 may provide a promising therapeutic tool for the prevention of DKD [62]. Moreover, Pentoxifylline (PTF) is a methylxanthine phosphodiesterase inhibitor with anti-inflammatory and anti-fibrotic properties, resulting in the downregulation of TNF-α [16]. In a study of diabetic patients with DKD, PTF reduced the level of albuminuria, displaying renoprotective properties [63].

## 4. Diabetes and Cardiovascular Complications: Oxidative Stress and Inflammation

Cardiovascular disease (CVD) remains the leading cause of premature death globally and poses a major health and economic burden worldwide [64,65]. CVD is a multifactorial disorder encompassing a broad range of injuries of the vasculature and heart including atherosclerosis, coronary heart disease, myocardial infarction, and cardiomyopathy [64,66]. Hypertension, dyslipidemia, obesity, insulin resistance, and chronic hyperglycemia often coexist and synergistically enhance the risk for CVD-related deaths. Studies suggest that diabetes increases the risk of atherosclerosis and myocardial infarction with diabetic patients demonstrating about a two times higher risk of CVD death than non-diabetic individuals [67]. Individuals with diabetes and kidney disease have an increased risk of CVD and premature death [67]. In fact, the presence of stage 3 kidney disease places a patient at a ten-fold greater risk of death, predominantly from cardiovascular disease, than the risk of progression to ESRD [68].

### 4.1. Atherosclerosis

The fundamental pathological mechanism underpinning macrovascular disease is the systemic narrowing of arterial walls, as a result of atherosclerosis [19]. Endothelial dysfunction initiates this process, allowing lipids to infiltrate the vessel wall where they are oxidized and exert a pro-inflammatory effect via cytokine secretion. This results in the recruitment of leukocytes and monocytes, which differentiate into macrophages and ultimately become foam cells, further producing cytokines and contributing to a self-perpetuating cycle of inflammation and atherosclerotic plaque formation. Over time, this plaque progressively thickens with the migration and proliferation of smooth muscle cells and collagen accumulation with eventual calcification forming an atherosclerotic, lipid rich lesion with a fibrous capsule. Plaque growth itself may obstruct the vessel lumen and impede vascular flow; however, disruption and rupture of this lesion is the key event which precipitates thrombus formation and acute vascular infarction [69]. The pathogenesis of atherosclerosis as described above is influenced throughout by oxidative stress and inflammation, with these pathways not only predisposing to development of atherosclerosis, but also promoting plaque rupture [70]. This is crucial in the context of diabetes, where increased production of ROS is prominent. Several ROS-producing systems are found in the walls of blood vessels: xanthine oxidase (XO), uncoupled endothelial nitric oxide synthase (eNOS), enzymes of the mitochondrial respiratory chain, and pro-oxidant enzymes NADPH oxidase (Nox) [71]. This entreats the question, how does oxidative stress lead to atherosclerosis and, hence, macrovascular complications?

### 4.2. Endothelial Dysfunction and Atherogenesis

Systemic oxidative stress associated with the diabetic state stimulates AGE production and accumulation of free radicals, causing direct damage to the endothelium, as well as increasing intravascular inflammation and leukocyte recruitment which further contributes to dysfunction and apoptosis of endothelial cells [72]. XO derived ROS also contribute to this endothelial dysfunction by generating superoxide and hydrogen peroxide in response to factors such as angiotensin II and oscillatory shear stress. Increased expression of XO has been demonstrated in human atherosclerotic plaque [73]. Furthermore, endothelial dysfunction is perpetuated by ROS induced uncoupling of eNOS. Nitric oxide (NO), primarily produced by eNOS, is crucial in physiological vasoprotection, by inducing vasodilation, inhibiting platelet aggregation and adhesion, and preventing atherogenesis [74]. However, excess superoxide rapidly inactivates NO and simultaneously renders eNOS dysfunctional, reducing NO bioavailability, further increasing superoxide production and hence predisposing to endothelial dysfunction [75].

Furthermore, prostaglandin–endoperoxide synthase, the cyclooxygenase (COX) enzymes, are responsible for the formation of prostanoids from arachidonic acid and mediate endothelial contraction and relaxation [26]. It has been demonstrated that elevated endothelial ROS can upregulate the expression of cyclooxygenase-2 (COX-2) resulting in the secretion of constrictive prostaglandins such as prostaglandin F_2α_. Expression and activity of COX-2 is shown to be enhanced in arteries of diabetic patients and this may contribute to the pathogenic cascade of diabetic endothelial dysfunction [26]. COX-2 also produces prostaglandin E_2_ (PGE_2_) which has also been linked to atherogenesis due to its association with dyslipidemia and the unresolving inflammatory state that leads to atherosclerotic lesion formation [76].

Notably, as a prominent source of ROS, the Nox enzymes are inevitably intertwined in the pathogenesis of atherosclerosis. However, in contrast to complications, such as diabetic nephropathy, Nox4 has been demonstrated to be atheroprotective [77]. Rather, it is Nox1 that appears to be most deleterious in the pathogenesis of atherosclerosis. For instance, global Nox1 deletion in mice was demonstrated to attenuate diabetes associated atherosclerosis [78]. Furthermore, when fed an atherogenic diet for 18 weeks, Nox1 deficient mice, displayed decreased atherosclerosis, macrophage infiltration and reduced lesion size at the aortic valve compared those where Nox1 was intact [75]. Similarly, in a diabetes-accelerated model of atherogenesis, deletion of Nox1 in ApoE knockout mice demonstrated reduced levels of vascular ROS production and were strongly protected from vascular inflammation and plaque development [78]. This suggests that the process of diabetic atherogenesis is contributed to by Nox1 and its derived ROS.

### 4.3. Inflammation and Lesion Progression

Vascular smooth muscle cells (VSMCs) contribute to an inflammatory cascade with the production of cytokines whilst also expressing adhesion molecules, including VCAM-1 and ICAM-1, that enhance the retention of cells within the lesion [79]. In addition, ROS and AGE promote each other bi-directionally, whilst both agents stimulate LDL oxidation, which in turn is known to stimulate MCP-1, ICAM-1, and VCAM-1 expression [80]. Moreover, hyperglycemia-related oxidative stress contributes to the activation of pro-inflammatory signaling pathways, including NFκB, mitogen-activated protein kinase (MAPK), or PKC, further perpetuating the progression of atherosclerosis [81]. ROS also mediate various signaling pathways that underlie lesion progression and ultimately plaque rupture [82]. For instance, AGE are able to promote the progression of atherosclerosis by inducing plaque calcification and their interaction with the receptor for AGE (RAGE) results in production of adhesion molecules and cytokines that perpetuate an inflammatory environment and enhance atherosclerotic lesion formation and progression [70,83]. Additionally, oxidative stress seen in diabetes contributes to platelet hyper-reactivity, thereby increasing thrombotic risk [84]. This platelet hyper-reactivity is enhanced by the prevalent combination of oxidative stress with hyperglycemia and elevated vascular shear stress in patients with DM. Crucially, these factors are not targeted by current anti-platelet agents, providing an explanatory avenue as to why patients with diabetes exhibit a poorer response to standard anti-platelet therapy [85]. Moreover, platelets themselves exhibit substantial inflammatory potential, expressing a plethora of cytokines and chemokines that enhance the recruitment of inflammatory cells to the site of the lesion and perpetuate the inflammatory milieu present in atherosclerosis [86]. It is clear that oxidative stress and inflammation are deeply involved in the development and progression of diabetic atherosclerotic disease, which then manifests in complications, including stroke and peripheral vascular disease. 

## 5. Therapeutic Approaches for Diabetes Associated CVD

### 5.1. Current Therapeutic Approaches in Diabetes Associated CVD

Current interventions for the management cardiovascular risk in diabetic patients principally includes the modification of lifestyle factors (such as changes in nutrition, smoking status, and level of physical activity), as well as the administration of antihypertensive and lipid-lowering medications [87].

#### 5.1.1. Antihypertensive Medication

As hypertension is a common comorbidity in those with T2DM and represents a crucial risk factor for cardiovascular disease, blood pressure control with an ARB or an ACEi is commonly initiated. Tight blood pressure control has associated with a reduction in the risk of diabetes related deaths and complications. Furthermore, as previously outlined, treatment with an ARB or ACEi is known to have positive effects on albuminuria and may reduce the risk of decline in kidney function [87].

#### 5.1.2. Lipid-Lowering Medication

Statins represent the first line pharmacological approach for dyslipidemia. Statin therapy is clearly of benefit in those with T2DM and high CVD risk, providing a significant decrease in coronary artery disease morbidity and mortality [88]. Evidence for other lipid reducing therapies is increasingly mounting. For example, combined administration of a statin and ezetimibe (a cholesterol-absorption inhibitor) in diabetic patients was associated with a reduced risk of coronary events and cardiovascular death, in comparison to statin therapy alone [89]. Fibrates (such as fenofibrate) also play a limited role, having demonstrated significant benefit toward diabetic retinopathy, as well as cardiovascular risk in patients with metabolic syndrome [90,91].

**Figure 2:**
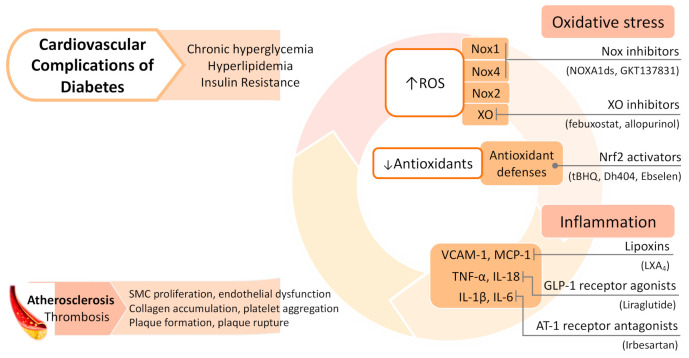
Pathophysiology and therapeutic strategies of diabetes associated cardiovascular disease. AT-1, angiotensin II type 1; GLP-1, glucagon-like peptide 1; IL, interleukin; MCP-1, monocyte chemotactic protein-1; Nox, NADPH oxidase; Nrf2, nuclear factor erythroid 2–related factor 2; ROS, reactive oxygen species; TNF-α, tumor necrosis factor alpha; VCAM-1, vascular cell adhesion protein 1; XO, xanthine oxidase.

Despite these current therapies, the disproportionate CVD burden in diabetic patients remains; hence, new pharmacological targets are being increasingly explored. Given the complex and multifaceted association between oxidative stress, inflammation, and diabetic cardiovascular complications, it follows that inhibition of these factors could prove to be effective clinical strategies to prevent and treat disease. Such strategies can be thought of in three main groups: (1) those that counteract with excess ROS accumulation (i.e., antioxidants), (2) those that inhibit the sources of ROS formation, and (3) those that inhibit the associated inflammatory pathways (Figure 2).

### 5.2. Therapeutic Approaches Counteracting Excess ROS Accumulation in Diabetes Associated CVD

#### 5.2.1. Antioxidants

As previously outlined, antioxidants are compounds that counterbalance ROS production, thereby alleviating oxidative stress. Nutritional antioxidant sources include vitamin E, C, and beta carotene [92]. However, randomized trials indicate that supplementation with these antioxidants, alone or in combination offer little to no overall benefit in the primary or secondary prevention of cardiovascular disease [93,94,95]. Moreover, the opposite extreme of redox balance, namely reductive stress, is known to cause tissue damage, including cardiac injury [96]. As a result, the focus has shifted from molecules that blindly target, or scavenge for, ROS to an emphasis on compounds that inhibit the enzymes responsible for ROS production.

#### 5.2.2. Nrf2 Activators

As previously mentioned, the protective effects of Nrf2 activation is being increasingly explored in the context of diabetic nephropathy. However, this therapeutic route is also highly relevant when tackling the cardiovascular complications of diabetes. For example, in animal models of type 1 diabetes, the Nrf2 activator Ebselen has been demonstrated to reduce oxidative stress and atherosclerosis [97,98]. Furthermore, the Nrf2 activator Dh404, was shown to significantly reduce endothelial dysfunction in diabetic mice. This effect was also associated with a downregulation of inflammatory markers, including ICAM-1, VCAM-1, and IL-1β, as well as decreased expression of Nox1 and Nox2 [44]. In contrary however, the Nrf2 activator bardoxolone methyl (BM) undertook the BEACON trial, but was terminated prematurely due to a significantly higher rate of cardiovascular events in the BM group, versus those receiving the placebo [99]. This effect was mitigated when controlling for patients with a higher baseline risk for heart failure, as determined by elevated B-type natriuretic peptide and past hospitalization for heart failure [100]. This may reflect the need for more restricted patient selection in future clinical trials. More recently, another Nrf2 activator named tert-butyl hydroquinone (tBHQ) has been investigated in the context of diabetic atherosclerosis. Administration of tBHQ in STZ-induced mouse models of diabetes resulted in a significant decrease in plaque extension, size and lipid content with a concurrent decrease in inflammation and chemokine expression [99]. Hence, Nrf2 activation remains a promising atheroprotective avenue in diabetes, due to its attenuating effect toward oxidative stress and inflammation.

### 5.3. Therapeutic Approaches Inhibiting ROS Production in Diabetes Associated CVD

#### 5.3.1. Nox Inhibitors

Administration of the dual Nox1/Nox4 inhibitor, GKT137831, in mice models upon induction of diabetes has been shown to attenuate the diabetes induced increases in atherosclerotic plaque area [78]. Furthermore, the use of GKT137831 as a delayed intervention, following established diabetic macrovascular disease, is of interest. In mice with diabetes and atherosclerosis, administration of GKT137831 at a dose of 30 mg/kg decreased aortic plaque area, compared to untreated mice. However, mice given an increased dosage of 60 mg/kg did not display amelioration in atherosclerosis yet were afforded a significant reno-protective effect. This is likely due to the protective nature of Nox4 in the macro-vasculature contrasted by its pathological action in the diabetic kidney disease [77]. Peptide based inhibitors are an alternate therapeutic strategy, with NOXA1ds being specifically designed to block NOX1. The hypoxia-induced or angiotensin II–induced production of ROS in endothelial and vascular smooth muscle cells, respectively, was significantly inhibited by NOXA1ds [101]. However, more research is needed in specific relation to atherosclerosis and cardiovascular disease. 

#### 5.3.2. XO Inhibitors

Another avenue aiming to reduce the production of ROS is inhibition of Xanthine oxidase. In ApoE−/− mice, it has been demonstrated that administration of the XO inhibitor Febuxostat reduces arterial ROS levels and endothelial dysfunction, thereby decreasing pro-inflammatory markers and attenuating histological features of atherosclerosis [102]. Similarly, inhibition of xanthine oxidoreductase (which includes XO and xanthine dehydrogenase) with oral allopurinol significantly ameliorated calcification and lipid accumulation in the aortas of ApoE−/− mice, with a concurrent reduction of inflammatory cytokines [103].

### 5.4. Therapeutic Approaches Inhibiting Inflammation in Diabetes Associated CVD

#### 5.4.1. Lipoxins

As inflammatory pathways contribute to the pathogenesis of diabetic complications, including atherosclerosis, it follows that resolution of this inflammatory state could be protective. For instance, lipoxin A4 (LXA4), is an endogenous mediator of inflammation [104]. Brennan et al, demonstrated that in diabetic ApoE−/− mice, treatment with LXA4 reduced expression of inflammatory markers including VCAM-1, MCP-1, IL-1β, and IL-6, whilst attenuating aortic plaque development. Importantly, administration of the drug was also atheroprotective in diabetic mice with established disease. This presents pharmacological mediation of inflammation as a promising therapeutic avenue in diabetic vascular complications [57].

#### 5.4.2. GLP-1 Receptor Agonists

Recent studies have also shed light on the neuroprotective effects of suppressing inflammation, in the setting of diabetic cerebrovascular disease [105]. Shi et al. determined that liraglutide, but not insulin, decreased infarct volume and improved neurological deficits due to cerebral ischemic injury in diabetic rats. This neuroprotective effect was therefore not simply mediated by a reduction in hyperglycemia, but likely due to the drugs ability to suppress inflammation and oxidative stress [106]. Similarly, diabetic rats with cerebral ischemia displayed significant amelioration in neurological deficits and reduction in infarct volume via reduction of oxidative stress, when pretreated with recombinant human glucagon–like peptide-1 (GLP-1) [107]. In fact, GLP-1-based therapies are neuroprotective in both diabetic and non-diabetic animals by significantly affecting inflammation, oxidative stress and apoptotic sequelae of stroke [108]. More recently, Giglio et al. demonstrated the beneficial effects of liraglutide on MicroRNAs in patients with T2DM. Such microRNAs play a role in cardiometabolic disease. Serum levels of microRNAs were increased after liraglutide treatment, independent of metabolic parameters, indicating that liraglutide may have a direct epigenetic effect toward maintaining endothelial cell homeostasis in patients with T2DM [109].

## 6. Conclusions

Globally, cardiovascular disease represents an immense source of morbidity and mortality in diabetic patients, with such individuals exhibiting a doubling of cardiovascular risk. Despite major advances in glucose-lowering treatments over the last decade, diabetes-associated cardiovascular and kidney disease continue to pose a heavy burden on the global healthcare system. One of the most important comorbidities that impacts the outcome and clinical management of cardiovascular disease in diabetes is renal disease, as reflected by proteinuria and declining renal function. Indeed, reduced estimated glomerular filtration rate is a potent predictor of cardiovascular mortality and complications. On the other hand, progressing cardiovascular injury can accelerate worsening of renal function in diabetes. Therefore, it appears that there is bidirectional interplay between cardiovascular and renal disease, as seen in diabetes. The precise underlying mechanisms of renal and cardiovascular disease are yet to be elucidated. However, it appears that, in diabetes, chronic hyperglycemia enhances the formation of ROS and activates mediators of inflammation, suppressing antioxidant defense mechanisms and ultimately contributing to oxidative stress, which leads to renal and cardiovascular vascular injury. In addition, a bidirectional relationship between ROS and the mediators of inflammation plays a crucial role in promoting renal and cardiovascular fibrosis in diabetes. Therefore, pharmacological agents targeting this vicious connection between ROS, inflammation, and fibrosis could represent a potential therapeutic option for the treatment and prevention of CVD and kidney disease in diabetes, as summarized in Table 1, below.

## Figures and Tables

**Table 1:** Pharmacological approaches toward the renal and cardiovascular complications of diabetes.

Current and Potential Therapies	DKD and CVD
RAAS Inhibitors	ACEi, ARBs [31,87]
SGLT-2 Inhibitors	Empagliflozin, Canaglifozin, Phlorizin [35,36,37]
Lipid-lowing medication	Statins, Fibrates [88,89,90,91]
Nrf2 Activators	Bardoxolone methyl, Sulforaphane, Ebselen, Dh404, tBHQ [45,46,97,98,99]
NADPH Oxidase Inhibitors	GKT137831, NOXA1ds [21,51,52,77,78,101]
XO Inhibitors	Allopurinol, Febuxostat [54,55,102,103]
Lipoxins	LXA_4_ [57,104]
NLRP3 Inhibitors	MCC950 [60,61,62,63]
GLP-1 Receptor Agonists	Liraglutide [106,109]

CVD, cardiovascular disease; DKD, diabetic kidney disease.

## References

[1] Zimmet P., Alberti K.G., Magliano D.J., Bennett P.H. (2016). Diabetes mellitus statistics on prevalence and mortality: Facts and fallacies. Nat. Rev. Endocrinol..

[2] (2019). IDF Diabetes Atlas.

[3] (2020). Diabetes in Australia. https://www.diabetesaustralia.com.au/diabetes-in-australia.

[4] Zimmet P.Z., Magliano D.J., Herman W.H., Shaw J.E. (2014). Diabetes: A 21st century challenge. Lancet Diabetes Endocrinol..

[5] Domingueti C.P., Dusse L.M.S., Carvalho M.D., De Sousa L.P., Gomes K.B., Fernandes A.P. (2016). Diabetes mellitus: The linkage between oxidative stress, inflammation, hypercoagulability and vascular complications. J. Diabetes Complicat..

[6] Teodoro J.S., Nunes S., Rolo A.P., Reis F., Palmeira C.M. (2019). Therapeutic options targeting oxidative stress, mitochondrial dysfunction and inflammation to hinder the progression of vascular complications of diabetes. Front. Physiol..

[7] Stitt A.W., Curtis T.M., Chen M., Medina R.J., McKay G.J., Jenkins A., Gardiner T.A., Lyons T.J., Hammes H.P., Simo R. (2016). The progress in understanding and treatment of diabetic retinopathy. Prog. Retinal Eye Res..

[8] Garber A.J., Handelsman Y., Grunberger G., Einhorn D., Abrahamson M.J., Barzilay J.I., Blonde L., Bush M.A., DeFronzo R.A., Garber J.R. (2020). Consensus statement by the American Association of Clinical Endocrinologists and American College of Endocrinology on the comprehensive type 2 diabetes management algorithm—2020 executive summary. Endocr. Pract..

[9] Herman W.H. (2017). The global burden of diabetes: An overview. Diabetes Mellitus in Developing Countries and Underserved Communities.

[10] Pickering R.J., Rosado C.J., Sharma A., Buksh S., Tate M., de Haan J.B. (2018). Recent novel approaches to limit oxidative stress and inflammation in diabetic complications. Clin. Transl. Immunol..

[11] Pizzino G., Irrera N., Cucinotta M., Pallio G., Mannino F., Arcoraci V., Squadrito F., Altavilla D., Bitto A. (2017). Oxidative stress: Harms and benefits for human health. Oxidative Med. Cell. Longev..

[12] Jha J.C., Banal C., Chow B.S., Cooper M.E., Jandeleit-Dahm K. (2016). Diabetes and kidney disease: Role of oxidative stress. Antioxid. Redox Signal..

[13] Oguntibeju O.O. (2019). Type 2 diabetes mellitus, oxidative stress and inflammation: Examining the links. Int. J. Physiol. Pathophysiol. Pharmacol..

[14] Calderon G., Juarez O., Hernandez G., Punzo S., de la Cruz Z. (2017). Oxidative stress and diabetic retinopathy: Development and treatment. Eye.

[15] Aghadavod E., Khodadadi S., Baradaran A., Nasri P., Bahmani M., Rafieian-Kopaei M. (2016). Role of oxidative stress and inflammatory factors in diabetic kidney disease. Iran. J. Kidney Dis..

[16] Jha J.C., Ho F., Dan C., Jandeleit-Dahm K. (2018). A causal link between oxidative stress and inflammation in cardiovascular and renal complications of diabetes. Clin. Sci..

[17] White S., Chadban S. (2014). Diabetic kidney disease in Australia: Current burden and future projections. Nephrology.

[18] Paul S., Ali A., Katare R. (2020). Molecular complexities underlying the vascular complications of diabetes mellitus—A comprehensive review. J. Diabetes Complicat..

[19] Fowler M.J. (2008). Microvascular and macrovascular complications of diabetes. Clin. Diabetes.

[20] Sifuentes-Franco S., Padilla-Tejeda D.E., Carrillo-Ibarra S., Miranda-Diaz A.G. (2018). Oxidative stress, apoptosis, and mitochondrial function in diabetic nephropathy. Int. J. Endocrinol..

[21] Jha J.C., Gray S.P., Barit D., Okabe J., El-Osta A., Namikoshi T., Thallas-Bonke V., Wingler K., Szyndralewiez C., Heitz F. (2014). Genetic targeting or pharmacologic inhibition of NADPH oxidase Nox4 provides renoprotection in long-term diabetic nephropathy. J. Am. Soc. Nephrol..

[22] Jha J.C., Thallas-Bonke V., Banal C., Gray S.P., Chow B.S.M., Ramm G., Quaggin S.E., Cooper M.E., Schmidt H.H., Jandeleit-Dahm K.A. (2016). Podocyte-specific Nox4 deletion affords renoprotection in a mouse model of diabetic nephropathy. Diabetologia.

[23] Holterman C.E., Thibodeau J.F., Towaij C., Gutsol A., Montezano A.C., Parks R.J., Cooper M.E., Touyz R.M., Kennedy C.R. (2014). Nephropathy and elevated BP in mice with podocyte-specific NADPH oxidase 5 expression. J. Am. Soc. Nephrol..

[24] Jha J.C., Banal C., Okabe J., Gray S.P., Hettige T., Chow B.S.M., Thallas-Bonke V., De Vos L., Holterman C.E., Coughlan M.T. (2017). NADPH oxidase nox5 accelerates renal injury in diabetic nephropathy. Diabetes.

[25] Jha J.C., Dai A., Holterman C.E., Cooper M.E., Touyz R.M., Kennedy C.R., Jandeleit-Dahm K.A.M. (2019). Endothelial or vascular smooth muscle cell-specific expression of human NOX5 exacerbates renal inflammation, fibrosis and albuminuria in the Akita mouse. Diabetologia.

[26] Tessaro F.H.G., Ayala T.S., Martins J.O. (2015). Lipid mediators are critical in resolving inflammation: A review of the emerging roles of eicosanoids in diabetes mellitus. BioMed Res. Int..

[27] Xue X., Ren J., Sun X., Gui Y., Feng Y., Shu B., Wei W., Lu Q., Liang Y., He W. (2018). Protein kinase Cα drives fibroblast activation and kidney fibrosis by stimulating autophagic flux. J. Biol. Chem..

[28] Thallas-Bonke V., Jha J.C., Gray S.P., Barit D., Haller H., Schmidt H.H.H.W., Coughlan M.T., Cooper M.E., Forbes J.M., Jandeleit-Dahm K.A.M. (2014). Nox-4 deletion reduces oxidative stress and injury by PKC-α-associated mechanisms in diabetic nephropathy. Physiol. Rep..

[29] Hu F., Xue M., Li Y., Jia Y.-J., Zheng Z.-J., Yang Y.-L., Guan M.-P., Sun L., Xue Y.-M. (2018). Early growth response 1 (Egr1) is a transcriptional activator of NOX4 in oxidative stress of diabetic kidney disease. J. Diabetes Res..

[30] Breyer M.D., Susztak K. (2016). The next generation of therapeutics for chronic kidney disease. Nat. Rev. Drug. Discov..

[31] Lozano-Maneiro L., Puente-García A. (2015). Renin-angiotensin-aldosterone system blockade in diabetic nephropathy. Present evidences. J. Clin. Med..

[32] Kawanami D., Matoba K., Takeda Y., Nagai Y., Akamine T., Yokota T., Sango K., Utsunomiya K. (2017). SGLT2 inhibitors as a therapeutic option for diabetic nephropathy. Int. J. Mol. Sci..

[33] Kruger D., Valentine V. (2020). Canagliflozin for the treatment of diabetic kidney disease and implications for clinical practice: A narrative review. Diabetes Ther..

[34] Neal B., Perkovic V., Mahaffey K.W., de Zeeuw D., Fulcher G., Erondu N., Shaw W., Law G., Desai M., Matthews D.R. (2017). Canagliflozin and cardiovascular and renal events in type 2 diabetes. N. Engl. J. Med..

[35] Wanner C., Inzucchi S.E., Lachin J.M., Fitchett D., von Eynatten M., Mattheus M., Johansen O.E., Woerle H.J., Broedl U.C., Zinman B. (2016). Empagliflozin and progression of kidney disease in type 2 diabetes. N. Engl. J. Med..

[36] Zinman B., Wanner C., Lachin J.M., Fitchett D., Bluhmki E., Hantel S., Mattheus M., Devins T., Johansen O.E., Woerle H.J. (2015). Empagliflozin, cardiovascular outcomes, and mortality in type 2 diabetes. N. Engl. J. Med..

[37] Osorio H., Coronel I., Arellano A., Pacheco U., Bautista R., Franco M., Escalante B. (2012). Sodium-glucose cotransporter inhibition prevents oxidative stress in the kidney of diabetic rats. Oxid. Med. Cell. Longev..

[38] Hsia D.S., Grove O., Cefalu W.T. (2017). An update on sodium-glucose co-transporter-2 inhibitors for the treatment of diabetes mellitus. Curr. Opin. Endocrinol. Diabetes Obes..

[39] Monami M., Nardini C., Mannucci E. (2014). Efficacy and safety of sodium glucose co-transport-2 inhibitors in type 2 diabetes: A meta-analysis of randomized clinical trials. Diabetes Obes. Metab..

[40] Patel A., MacMahon S., Chalmers J., Neal B., Billot L., Woodward M., Marre M., Cooper M., Glasziou P., Grobbee D. (2008). Intensive blood glucose control and vascular outcomes in patients with type 2 diabetes. N. Engl. J. Med..

[41] Galeshkalami N.S., Abdollahi M., Najafi R., Baeeri M., Jamshidzade A., Falak R., Gholami M.D., Hassanzadeh G., Mokhtari T., Hassani S. (2019). Alpha-lipoic acid and coenzyme Q10 combination ameliorates experimental diabetic neuropathy by modulating oxidative stress and apoptosis. Life Sci..

[42] Tavafi M. (2013). Diabetic nephropathy and antioxidants. J. Nephropathol..

[43] Shelton P., Jaiswal A.K. (2013). The transcription factor NF-E2-related factor 2 (Nrf2): A protooncogene?. FASEB J..

[44] Sharma A., Rizky L., Stefanovic N., Tate M., Ritchie R.H., Ward K.W., de Haan J.B. (2017). The nuclear factor (erythroid-derived 2)-like 2 (Nrf2) activator dh404 protects against diabetes-induced endothelial dysfunction. Cardiovasc. Diabetol..

[45] Choi B.-H., Kang K.-S., Kwak M.-K. (2014). Effect of redox modulating NRF2 activators on chronic kidney disease. Molecules.

[46] Ruiz S., Pergola P.E., Zager R.A., Vaziri N.D. (2013). Targeting the transcription factor Nrf2 to ameliorate oxidative stress and inflammation in chronic kidney disease. Kidney Int..

[47] Altenhöfer S., Radermacher K.A., Kleikers P.W.M., Wingler K., Schmidt H.H.H.W. (2015). Evolution of NADPH oxidase inhibitors: Selectivity and mechanisms for target engagement. Antioxid. Redox Signal..

[48] Sedeek M., Callera G., Montezano A., Gutsol A., Heitz F., Szyndralewiez C., Page P., Kennedy C.R.J., Burns K.D., Touyz R.M. (2010). Critical role of Nox4-based NADPH oxidase in glucose-induced oxidative stress in the kidney: Implications in type 2 diabetic nephropathy. Am. J. Physiol. Renal Physiol..

[49] Sedeek M., Gutsol A., Montezano A.C., Burger D., Nguyen Dinh Cat A., Kennedy C.R., Burns K.D., Cooper M.E., Jandeleit-Dahm K., Page P. (2013). Renoprotective effects of a novel Nox1/4 inhibitor in a mouse model of Type 2 diabetes. Clin. Sci..

[50] Casas A.I., Dao V.T., Daiber A., Maghzal G.J., Di Lisa F., Kaludercic N., Leach S., Cuadrado A., Jaquet V., Seredenina T. (2015). Reactive oxygen-related diseases: Therapeutic targets and emerging clinical indications. Antioxid. Redox Signal..

[51] Sewell J. (2019). Global Engage. https://www.global-engage.com/life-science/inhibiting-nox-enzymes-to-treat-multiple-diseases-with-high-medical-need/.

[52] Reutens A.T., Jandeleit-Dahm K., Thomas M., Salim A., de Livera A.M., Bach L.A., Colman P.G., Davis T.M., Ekinci E.I., Fulcher G. (2020). A physician-initiated double-blind, randomised, placebo-controlled, phase 2 study evaluating the efficacy and safety of inhibition of NADPH oxidase with the first-in-class Nox-1/4 inhibitor, GKT137831, in adults with type 1 diabetes and persistently elevated urinary albumin excretion: Protocol and statistical considerations. Contemp. Clin. Trials.

[53] Almeer R.S., Hammad S.F., Leheta O.F., Abdel Moneim A.E., Amin H.K. (2019). Anti-inflammatory and anti-hyperuricemic functions of two synthetic hybrid drugs with dual biological active sites. Int. J. Mol. Sci..

[54] Kosugi T., Nakayama T., Heinig M., Zhang L., Yuzawa Y., Sanchez-Lozada L.G., Roncal C., Johnson R.J., Nakagawa T. (2009). Effect of lowering uric acid on renal disease in the type 2 diabetic db/db mice. Am. J. Physiol. Renal Physiol..

[55] Sircar D., Chatterjee S., Waikhom R., Golay V., Raychaudhury A., Chatterjee S., Pandey R. (2015). Efficacy of febuxostat for slowing the GFR decline in patients with CKD and asymptomatic hyperuricemia: A 6-month, double-blind, randomized, placebo-controlled trial. Am. J. Kidney Dis..

[56] Mukri M.N.A., Kong W.-Y., Mustafar R., Shaharir S.S., Shah S.A., Abdul Gafor A.H., Mohd R., Abdul Cader R., Kamaruzaman L. (2018). Role of febuxostat in retarding progression of diabetic kidney disease with asymptomatic hyperuricemia: A 6-months open-label, randomized controlled trial. EXCLI J..

[57] Brennan E.P., Mohan M., McClelland A., de Gaetano M., Tikellis C., Marai M., Crean D., Dai A., Beuscart O., Derouiche S. (2018). Lipoxins Protect Against Inflammation in Diabetes-Associated Atherosclerosis. Diabetes.

[58] Wu J., Ding D.H., Li Q.Q., Wang X.Y., Sun Y.Y., Li L.J. (2019). Lipoxin A4 regulates lipopolysaccharide-induced BV2 microglial activation and differentiation via the notch signaling pathway. Front. Cell. Neurosci..

[59] Zhou Y., You H., Zhang A.J., Jiang X.L., Pu Z.Y., Xu G.Q., Zhao M. (2020). Lipoxin A4 attenuates uric acid‑activated, NADPH oxidase‑dependent oxidative stress by interfering with translocation of p47phox in human umbilical vein endothelial cells. Exp. Ther. Med..

[60] Van der Heijden T., Kritikou E., Venema W., van Duijn J., van Santbrink P.J., Slütter B., Foks A.C., Bot I., Kuiper J. (2017). NLRP3 inflammasome inhibition by MCC950 reduces atherosclerotic lesion development in apolipoprotein E–deficient mice—brief report. Arterioscler. Thromb. Vasc. Biol..

[61] Krishnan S.M., Ling Y.H., Huuskes B.M., Ferens D.M., Saini N., Chan C.T., Diep H., Kett M.M., Samuel C.S., Kemp-Harper B.K. (2019). Pharmacological inhibition of the NLRP3 inflammasome reduces blood pressure, renal damage, and dysfunction in salt-sensitive hypertension. Cardiovasc. Res..

[62] Zhang C., Zhu X., Li L., Ma T., Shi M., Yang Y., Fan Q. (2019). A small molecule inhibitor MCC950 ameliorates kidney injury in diabetic nephropathy by inhibiting NLRP3 inflammasome activation. Diabetes Metab. Syndr. Obes..

[63] Navarro-González J.F., Mora-Fernández C., Muros de Fuentes M., Chahin J., Méndez M.L., Gallego E., Macía M., del Castillo N., Rivero A., Getino M.A. (2015). Effect of pentoxifylline on renal function and urinary albumin excretion in patients with diabetic kidney disease: The PREDIAN trial. J. Am. Soc. Nephrol..

[64] Benjamin E.J., Blaha M.J., Chiuve S.E., Cushman M., Das S.R., Deo R., De Ferranti S.D., Floyd J., Fornage M., Gillespie C. (2017). Heart disease and stroke statistics—2017 update. Circulation.

[65] Haffner S.M., Lehto S., R&nnemaa T., Py&r&l& K., Laakso M. (1998). Mortality from coronary heart disease in subjects with type 2 diabetes and in nondiabetic subjects with and without prior myocardial infarction. N. Engl. J. Med..

[66] Reaven G.M., Chen Y.I. (1996). Insulin resistance, its consequences, and coronary heart disease: Must we choose one culprit?. Circulation.

[67] Fox C.S., Matsushita K., Woodward M., Bilo H.J., Chalmers J., Heerspink H.J.L., Lee B.J., Perkins R.M., Rossing P., Sairenchi T. (2012). Associations of kidney disease measures with mortality and end-stage renal disease in individuals with and without diabetes. Lancet.

[68] Pálsson R., Patel U.D. (2014). Cardiovascular complications of diabetic kidney disease. Adv. Chronic. Kidney Dis..

[69] Fernando S., Bursill C.A., Nicholls S.J., Psaltis P.J., Fitridge R. (2020). Pathophysiology of atherosclerosis. Mechanisms of Vascular Disease: A Textbook for Vascular Specialists.

[70] Wang Z.-Q., Jing L.-L., Yan J.-C., Sun Z., Bao Z.-Y., Shao C., Pang Q.-W., Geng Y., Zhang L.-L., Li L.-H. (2018). Role of AGEs in the progression and regression of atherosclerotic plaques. Glycoconj. J..

[71] Yang X., Li Y., Li Y., Ren X., Zhang X., Hu D., Gao Y., Xing Y., Shang H. (2017). Oxidative stress-mediated atherosclerosis: Mechanisms and therapies. Front. Physiol..

[72] Severino P., D’Amato A., Netti L., Pucci M., Infusino F., Maestrini V., Mancone M., Fedele F. (2019). Myocardial Ischemia and Diabetes Mellitus: Role of Oxidative Stress in the Connection between Cardiac Metabolism and Coronary Blood Flow. J. Diabetes Res..

[73] Kattoor A.J., Pothineni N.V.K., Palagiri D., Mehta J.L. (2017). Oxidative stress in atherosclerosis. Curr. Atheroscler. Rep..

[74] Li H., Horke S., Förstermann U. (2013). Oxidative stress in vascular disease and its pharmacological prevention. Trends Pharmacol. Sci..

[75] Shimizu H., Nakagawa Y., Murakami C., Aoki N., Kim-Mitsuyama S., Miyazaki H. (2010). Protein tyrosine phosphatase PTPεM negatively regulates PDGF β-receptor signaling induced by high glucose and PDGF in vascular smooth muscle cells. Am. J. Physiol. Physiol..

[76] Nasrallah R., Hassouneh R., Hébert R.L. (2016). PGE2, Kidney Disease, and Cardiovascular Risk: Beyond Hypertension and Diabetes. J. Am. Soc. Nephrol..

[77] Gray S.P., Jha J.C., Kennedy K., van Bommel E., Chew P., Szyndralewiez C., Touyz R.M., Schmidt H., Cooper M.E., Jandeleit-Dahm K.A.M. (2017). Combined NOX1/4 inhibition with GKT137831 in mice provides dose-dependent reno- and atheroprotection even in established micro- and macrovascular disease. Diabetologia.

[78] Gray S.P., di Marco E., Okabe J., Szyndralewiez C., Heitz F., Montezano A.C., de Haan J.B., Koulis C., El-Osta A., Andrews K.L. (2013). NADPH oxidase 1 plays a key role in diabetes mellitus–accelerated atherosclerosis. Circulation.

[79] Gregersen I., Halvorsen B. (2017). Inflammatory mechanisms in atherosclerosis. Atherosclerosis-Yesterday, Today and Tomorrow.

[80] Leiva E., Wehinger S., Guzmán L., Orrego R. (2015). Role of oxidized LDL in atherosclerosis. Hypercholesterolemia.

[81] Arcambal A., Taïlé J., Rondeau P., Viranaïcken W., Meilhac O., Gonthier M.-P. (2019). Hyperglycemia modulates redox, inflammatory and vasoactive markers through specific signaling pathways in cerebral endothelial cells: Insights on insulin protective action. Free Radic. Biol. Med..

[82] Madamanchi N.R., Vendrov A., Runge M.S. (2005). Oxidative stress and vascular disease. Arterioscler. Thromb. Vasc. Biol..

[83] Jandeleit-Dahm K., Watson A., Soro-Paavonen A. (2008). The age/rage axis in diabetes-accelerated atherosclerosis. Clin. Exp. Pharmacol. Physiol..

[84] Gaiz A., Mosawy S., Colson N., Singh I. (2017). Thrombotic and cardiovascular risks in type two diabetes; Role of platelet hyperactivity. Biomed. Pharmacother..

[85] Arthur J.F., Jandeleit-Dahm K., Andrews R.K. (2017). Platelet hyperreactivity in diabetes: Focus on GPVI signaling—Are useful drugs already available?. Diabetes.

[86] Lievens D., von Hundelshausen P. (2011). Platelets in atherosclerosis. Thromb. Haemost..

[87] Arnolda L.F. (2012). Guidelines for the Management of Absolute Cardiovascular Disease Risk.

[88] Kearney P.M., Blackwell L., Collins R., Keech A., Simes J., Peto R., Armitage J., Baigent C. (2008). Efficacy of cholesterol-lowering therapy in 18,686 people with diabetes in 14 randomised trials of statins: A meta-analysis. Lancet.

[89] Giugliano R.P., Cannon C.P., Blazing M.A., Nicolau J.C., Corbalán R., Špinar J., Park J.-G., White J.A., Bohula E.A., Braunwald E. (2018). Benefit of Adding Ezetimibe to Statin Therapy on Cardiovascular Outcomes and Safety in Patients With Versus Without Diabetes Mellitus. Circulation.

[90] Hermans M.P. (2010). Impact of fenofibrate on Type 2 diabetes patients with features of the metabolic syndrome: Subgroup analysis from FIELD. Curr. Cardiol. Rev..

[91] Kim N.H., Kim S.G. (2020). Fibrates revisited: Potential role in cardiovascular risk reduction. Diabetes Metab. J..

[92] Liu Z., Ren Z., Zhang J., Chuang C.-C., Kandaswamy E., Zhou T., Zuo L. (2018). Role of ROS and Nutritional Antioxidants in Human Diseases. Front. Physiol..

[93] Jenkins D.J.A., Spence J.D., Giovannucci E.L., Kim Y.-I., Josse R., Vieth R., Blanco Mejia S., Viguiliouk E., Nishi S., Sahye-Pudaruth S. (2018). Supplemental Vitamins and Minerals for CVD Prevention and Treatment. J. Am. Coll. Cardiol..

[94] Omenn G.S., Goodman G.E., Thornquist M.D., Balmes J., Cullen M.R., Glass A., Keogh J.P., Meyskens F.L., Valanis B., Williams J.H. (1996). Effects of a combination of beta carotene and vitamin A on lung cancer and cardiovascular disease. N. Engl. J. Med..

[95] Rapola J.M., Virtamo J., Ripatti S., Huttunen J.K., Albanes D., Taylor P.R., Heinonen O.P. (1997). Randomised trial of alpha-tocopherol and beta-carotene supplements on incidence of major coronary events in men with previous myocardial infarction. Lancet.

[96] Korge P., Calmettes G., Weiss J.N. (2015). Increased reactive oxygen species production during reductive stress: The roles of mitochondrial glutathione and thioredoxin reductases. Biochimica Biophysica Acta Bioenerg..

[97] Chew P., Yuen D.Y., Koh P., Stefanovic N., Febbraio M.A., Kola I., Cooper M.E., de Haan J.B. (2009). Site-specific antiatherogenic effect of the antioxidant ebselen in the diabetic apolipoprotein E–deficient mouse. Arterioscler. Thromb. Vasc. Biol..

[98] Chew P., Yuen D.Y., Stefanovic N., Pete J., Coughlan M.T., Jandeleit-Dahm K.A., Thomas M.C., Rosenfeldt F., Cooper M.E., de Haan J.B. (2010). Antiatherosclerotic and renoprotective effects of ebselen in the diabetic apolipoprotein E/GPx1-double knockout mouse. Diabetes.

[99] De Zeeuw D., Akizawa T., Audhya P., Bakris G.L., Chin M., Christ-Schmidt H., Goldsberry A., Houser M., Krauth M., Lambers Heerspink H.J. (2013). Bardoxolone Methyl in Type 2 Diabetes and Stage 4 Chronic Kidney Disease. N. Engl. J. Med..

[100] Chin M.P., Bakris G.L., Block G.A., Chertow G.M., Goldsberry A., Inker L.A., Heerspink H.J.L., O'Grady M., Pergola P.E., Wanner C. (2018). Bardoxolone Methyl Improves Kidney Function in Patients with Chronic Kidney Disease Stage 4 and Type 2 Diabetes: Post-Hoc Analyses from Bardoxolone Methyl Evaluation in Patients with Chronic Kidney Disease and Type 2 Diabetes Study. Am. J. Nephrol..

[101] Zhang Y., Murugesan P., Huang K., Cai H. (2020). NADPH oxidases and oxidase crosstalk in cardiovascular diseases: Novel therapeutic targets. Nat. Rev. Cardiol..

[102] Nomura J., Busso N., Ives A., Matsui C., Tsujimoto S., Shirakura T., Tamura M., Kobayashi T., So A., Yamanaka Y. (2014). Xanthine oxidase inhibition by febuxostat attenuates experimental atherosclerosis in mice. Sci. Rep..

[103] Kushiyama A., Okubo H., Sakoda H., Kikuchi T., Fujishiro M., Sato H., Kushiyama S., Iwashita M., Nishimura F., Fukushima T. (2012). Xanthine oxidoreductase is involved in macrophage foam cell formation and atherosclerosis development. Arterioscler. Thromb. Vasc. Biol..

[104] Liu X., Wang X., Duan X., Poorun D., Xu J., Zhang S., Gan L., He M., Zhu K., Ming Z. (2017). Lipoxin A4 and its analog suppress inflammation by modulating HMGB1 translocation and expression in psoriasis. Sci. Rep..

[105] Liu P., Zhao H., Wang R., Wang P., Tao Z., Gao L., Yan F., Liu X., Yu S., Ji X. (2015). MicroRNA-424 protects against focal cerebral ischemia and reperfusion injury in mice by suppressing oxidative stress. Stroke.

[106] Shi N., He J., Guo Q., Liu T., Han J. (2019). Liraglutide protects against diabetes mellitus complicated with focal cerebral ischemic injury by activating mitochondrial ATP-sensitive potassium channels. NeuroReport.

[107] Fang Y., Jiang D., Wang Y., Wang Q., Lv D., Liu J., Liu C. (2018). Neuroprotection of rhGLP-1 in diabetic rats with cerebral ischemia/reperfusion injury via regulation of oxidative stress, EAAT2, and apoptosis. Drug Dev. Res..

[108] Marlet I.R., Ölmestig J.N.E., Vilsbøll T., Rungby J., Kruuse C. (2018). Neuroprotective mechanisms of glucagon-like peptide-1-based therapies in ischaemic stroke: A systematic review based on pre-clinical studies. Basic Clin. Pharmacol. Toxicol..

[109] Giglio R.V., Nikolic D., Volti G.L., Stoian A.P., Banerjee Y., Magan-Fernandez A., Castellino G., Patti A.M., Chianetta R., Castracani C.C. (2020). Liraglutide Increases Serum Levels of MicroRNA-27b, -130a and -210 in Patients with Type 2 Diabetes Mellitus: A Novel Epigenetic Effect. Metabolites.

